# Chitosan-Genipin Microspheres for the Controlled Release of Drugs: Clarithromycin, Tramadol and Heparin

**DOI:** 10.3390/md8061750

**Published:** 2010-05-26

**Authors:** Ruth Harris, Elena Lecumberri, Angeles Heras

**Affiliations:** Instituto de Estudios Biofuncionales, Departamento Química-Física II. Universidad Complutense Paseo Juan XXIII, 128040, Madrid, Spain; E-Mails: ruthharris@ieb.ucm.es (R.H.); elena.lecumberri@gmail.com (E.L.)

**Keywords:** chitosan, genipin, microspheres, controlled release, clarithromycin, tramadol, heparin

## Abstract

The aim of this study was to first evaluate whether the chitosan hydrochloride-genipin crosslinking reaction is influenced by factors such as time, and polymer/genipin concentration, and second, to develop crosslinked drug loaded microspheres to improve the control over drug release. Once the crosslinking process was characterized as a function of the factors mentioned above, drug loaded hydrochloride chitosan microspheres with different degrees of crosslinking were obtained. Microspheres were characterized in terms of size, morphology, drug content, surface charge and capacity to control *in vitro* drug release. Clarithromycin, tramadol hydrochloride, and low molecular weight heparin (LMWH) were used as model drugs. The obtained particles were spherical, positively charged, with a diameter of 1–10 μm. X-Ray diffraction showed that there was an interaction of genipin and each drug with chitosan in the microspheres. In relation to the release profiles, a higher degree of crosslinking led to more control of drug release in the case of clarithromycin and tramadol. For these drugs, optimal release profiles were obtained for microspheres crosslinked with 1 mM genipin at 50 ºC for 5 h and with 5 mM genipin at 50 ºC for 5 h, respectively. In LMWH microspheres, the best release profile corresponded to 0.5 mM genipin, 50 ºC, 5 h. In conclusion, genipin showed to be eligible as a chemical-crosslinking agent delaying the outflow of drugs from the microspheres. However, more studies *in vitro* and *in vivo* must be carried out to determine adequate crosslinking conditions for different drugs.

## 1. Introduction

Among others, marine organisms continue to attract significant interest as potential sources of compounds with beneficial pharmacological activities. Among marine origin compounds, chitosan, a biopolymer obtained as a result of deacetylation of chitin, mainly obtained from crustacean exoeskeleton, has gained interest as a biocompatible and biodegradable matrix for drug controlled release and has been the subject of numerous studies in the last decades [1]. The hydrosoluble chitosan derivative, chitosan hydrochloride, was approved by Pharmacopea Europaea in 2002.

The use of crosslinking agents is considered a relevant tool to improve the control in drug delivery. Among other reagents, glutaraldehyde and tripolyphosphate have been widely used to crosslink biodegradable polymers. However, there are concerns over the toxicity of the most studied cross-linking agents, especially glutaraldehyde, which may impair the biocompatibility of the chitosan delivery system [2], and therefore, there is a need to provide a crosslinking agent that has low cytotoxicity.

**Figure 1:**
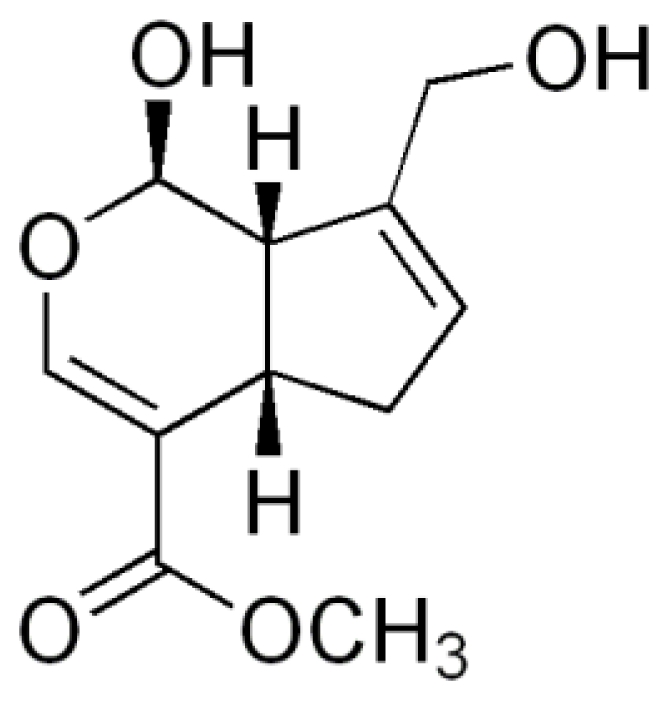
The chemical structure of genipin.

Geniposide is extracted from the fruit of *Gardenia jasminoides* Ellis, traditionally used in oriental medicine for the treatment of inflammation, headache, and hepatic disorders among others [3]. Its aglycone, genipin (Figure 1), has recently attracted much interest as a crosslinking agent for biomedical use because of its biocompatibility and because it forms stable and biocompatible crosslinked products [4,5]. The formation of crosslinks between genipin and primary amine groups [5] and the finding that genipin-crosslinked networks are significantly less cytotoxic than those crosslinked by glutaraldehyde [6] prompted us to use this chemical as a crosslinking agent to develop chitosan microspheres for controlled drug release.

In this study, the influence of some conditions (time, genipin concentration) over the crosslinking reaction was studied. Once the crosslinking process was characterized, microspheres with different degree of crosslinkage were obtained. Three different drugs, a macrolid antibiotic, clarithromycin, an analgesic drug, tramadol hydrochloride, and low molecular weight heparin (LMWH), were used as model drugs. Microspheres were studied in terms of size, morphology, drug content, surface charge and capacity to control drug release.

## 2. Results and Discussion

### 2.1. Characterization of the crosslinking reaction

In this study, genipin, a natural crosslinking agent, was used to crosslink chitosan microspheres to evaluate its effect on drug release. Genipin reacts with compounds containing primary amine groups, such as chitosan and some peptides and polypeptides, to form covalently crosslinked networks [7–10]. In the case of chitosan, genipin reacts with the free amino groups present in the glucosamine units. It has been reported that two separate reactions lead to the crosslinking between these two compounds [5]. The reaction between chitosan hydrochloride and genipin in solution was characterized by UV spectra analysis before spray-drying. The influence of reaction time and genipin concentration on the crosslinking reaction was studied.

**Figure 2:**
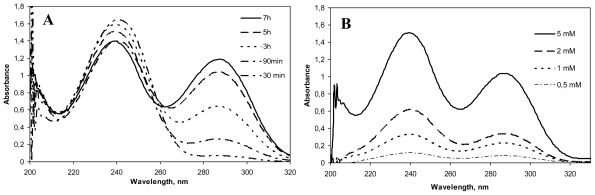
(A). UV spectra of Chitosan hydrochloride (HCS) (5 mg/mL)–genipin (Gnp) (5 mM) solutions incubated at 50 ºC for different crosslinking periods (30 min–7 h). (B). UV spectra of HCS (5 mg/mL)–Gnp solutions incubated at 50 ºC for 5 h with different concentrations of genipin (0.5, 1, 2 and 5 mM).

Genipin dissolved in water displayed an absorption peak at 240 nm, which was higher with increased genipin concentrations. After the addition of chitosan to the genipin solution, the absorption at 240 nm decreased and a new absorption peak at 290 could be seen. This absorption peak increased with reaction time and genipin concentration (Figure 2). Mi and co-workers [7] described that this decrease at 240 nm was due to the conversion of the ester group of genipin into an amide linkage formed after the nucleophilic attack by chitosan amino groups. On the other hand, the increase at 290 nm was attributed to the formation of the heterocyclic genipin-chitosan compound [11]. The presence of this peak and its increase with reaction time and genipin concentration is therefore an indication of the extent of the crosslinking between genipin and chitosan.

The effect of increasing the concentration of genipin on the crosslinking reaction at fixed temperature, 50 ºC (based on previous studies carried out in our laboratory) and time (5 h), was evaluated. In the same way, different reaction times (0–30 min) at 50 ºC and fixed genipin concentration (5 mM) were studied in relation to the crosslinking process. As shown in Figure 2, both increasing genipin concentration and reaction time led to a higher degree of crosslinking. For biomedical applications, the adequate crosslinking conditions should be studied in each case depending on the drug, location of release within the human body, and pharmacokinetic parameters, to achieve optimal controlled release.

### 2.2. Scanning electron microscopy (SEM)

**Figure 3:**
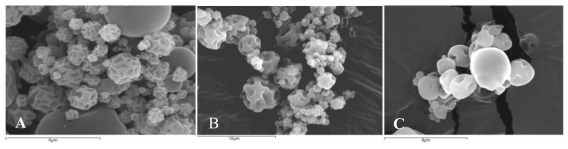
Scanning electron micrographs of chitosan hydrochloride microspheres. (A). Chitosan microspheres loaded with 50% (w/w) clarithromycin crosslinked with 1 mM genipin for 5 h at 50 ºC. (B). Chitosan microspheres loaded with 30% (w/w) tramadol hydrochloride crosslinked with 2 mM genipin for 5 h at 50 ºC. (C). Chitosan microspheres loaded with 10% (w/w) LMWH crosslinked with 0.5 mM genipin at 50 ºC for 5 h.

Figure 3 shows the morphology of chitosan microspheres crosslinked with genipin and loaded with clarithromycin (Figure 3A), tramadol hydrochloride (Figure 3B) or LMWH (Figure 3C) obtained by SEM. As can be seen, <10 μm microspheres were obtained in all cases. The obtained microspheres presented a smooth surface with indentations as a result of rapid particle shrinking during the drying process [12].

### 2.3. Zeta potential

The zeta potential of the microspheres is shown in Table 1 (microspheres loaded with clarithromycin), Table 2 (microspheres loaded with tramadol hydrochloride) and Table 3 (microspheres loaded with LMWH).

As can be seen with these three cases, the zeta potential values decreased when the microspheres were crosslinked with genipin, proving the genipin-polymer interaction. Nevertheless, all the microspheres showed positive charge, so all of them maintained the chitosan mucoadhesive and absorption enhancement properties and can be considered as potential drug release vehicles [13].

### 2.4. Drug-polymer interaction

**Figure 4:**
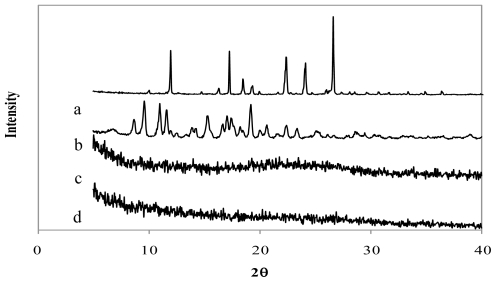
X-ray diffraction patterns of (a) genipin, (b) clarithromycin, (c) chitosan hydrochloride, (d) chitosan hydrochloride (5 mg/mL) microspheres loaded with clarithromycin (50% w/w) and crosslinked with genipin (1 mM) at 50 ºC for 5 h.

**Figure 5:**
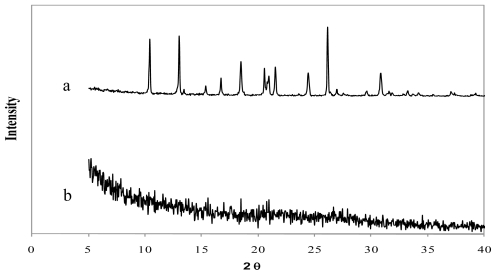
X-ray diffraction patterns of (a) tramadol hydrochloride and (b) chitosan hydrochloride (5 mg/mL) microspheres loaded with tramadol hydrochloride (30% w/w) and crosslinked with genipin (2 mM) at 50 ºC for 5 h.

**Figure 6:**
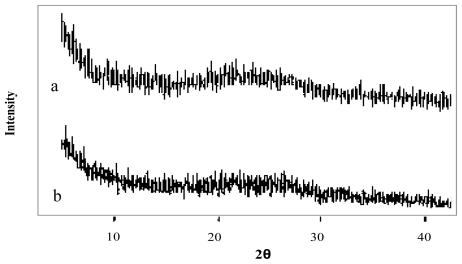
X-ray diffraction patterns of (a) low molecular weight heparin, LMWH (b) chitosan hydrochloride (1 mg/mL) microspheres loaded with LMWH (10% w/w) and crosslinked with genipin (0.5 mM) at 50 ºC for 5 h.

The X-Ray diffraction patterns of genipin, chitosan hydrochloride, assayed drugs, and the combination of all microsphere components are shown in Figures 4–6. Genipin itself, as well as clarithromycin and tramadol hydrochloride, showed high crystallinity. On the other hand, the chitosan hydrochloride X-ray pattern showed its amorphous structure. Genipin, as well as clarithromycin (Figure 4) and tramadol hydrochloride (Figure 5) are included in the amorphous structure of chitosan when incorporated in microspheres. As can be seen in Figure 6, the LMWH X-ray pattern shows it as an amorphous compound. LMWH loaded microspheres crosslinked with genipin show an X-ray diffraction pattern with no marked peaks, which indicates that the crosslinking agent is molecularly dispersed in the polymer matrix [14,15], which should help to retard the delivery of the drug [16].

### 2.5. Drug release studies

As mentioned in the experimental section, preliminary studies using different times, temperatures, and genipin concentrations were carried out for assayed drugs. 5 h and 50 ºC were revealed as adequate time and temperature conditions to achieve the desired degree of crosslinking. The genipin concentration range used for each drug is also a consequence of these previous results (data not shown).

The encapsulation efficiency was >85% for clarithromycin, >90% for tramadol, and 15–30% depending on the crosslinking degree (the higher the crosslinking degree the lesser the encapsulation efficiency) in the case of LMWH.

#### 2.5.1. Clarithromycin

**Figure 7:**
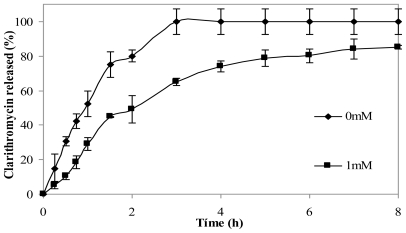
Release profiles of clarithromycin in SGF from chitosan hydrochloride (5 mg/mL) microspheres loaded with clarithromycin (50% w/w) without genipin (0 mM) and crosslinked with 1mM genipin at 50 ºC for 5 h.

The effect of crosslinking microspheres with genipin on clarithromycin release in simulated gastric fluid (SGF) was studied. The addition of the crosslinking agent led to the slower release of the drug. Figure 7 shows clarithromycin release profiles of non crosslinked chitosan microspheres and from microspheres crosslinked with 1 mM genipin for 5 h at 50 ºC. After 3 h, microspheres without genipin had released 100% of the drug, while crosslinked microspheres had released 60% of clarithromycin. The release from microspheres crosslinked with 0.5 mM genipin did not show significant differences with microspheres with 1mM genipin (results not shown).

#### 2.5.2. Tramadol hydrochloride

**Figure 8:**
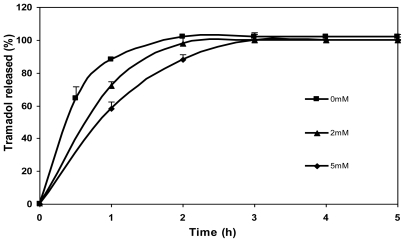
Release profiles of tramadol hydrochloride (30% w/w) in SGF from chitosan hydrochloride (5 mg/mL) microspheres without genipin (0 mM) and crosslinked with 2 mM and 5 mM genipin at 50 ºC for 5 h.

The effect of genipin concentration (0 mM, 2 mM and 5 mM) on the release of tramadol hydrochloride from chitosan microspheres is shown in Figure 8. As can be seen, drug release was slower in the case of microspheres crosslinked with genipin in comparison with the non crosslinked ones (0 mM). What is more, the tramadol release rate slowed down with increasing genipin concentration (5 mM genipin microspheres released the drug slower than 2 mM genipin microspheres). In the case of non crosslinked microspheres, a burst effect was observed during the initial hour (>80% of drug) and the total amount of drug encapsulated was released after 2 h. In contrast, the release of drug during the first hour from crosslinked microspheres was significantly lower. The amount of drug released after 2 h from microspheres crosslinked with genipin of different concentrations, 2 and 5 mM, was 97 and 88%, respectively, and it was completely released after 3 h. Therefore, as shown in preliminary studies, crosslinking with genipin controls the release of tramadol hydrochloride [17].

#### 2.5.3. Low molecular weight heparin, LMWH

Chitosan hydrochloride itself acted as a vehicle for controlled release of LMWH. The electrostatic attraction between chitosan and LMWH [18] slowed down the release of LMWH from the formed polyelectrolite. In addition, in this case, the release medium, PBS pH 7.4, contributed to delaying the drug release due to the reduced solubility of chitosan hydrochloride at this pH. Thus, in non crosslinked microspheres (HB), the delay in the release of the drug was the result of the formation of a chitosan-LMWH polyelectrolite together with reduced solubility of chitosan at release medium pH. In the HB release profile, an initial burst effect was observed followed by a more controlled release. This is in accordance with a high number of studies that revealed the fastest drug release from chitosan microspheres at the initial release stages [19]. After 30 minutes, 40% of the drug had been released from the microspheres. In the next 3 h, LMWH in the media reached 70% of the total incorporated in microspheres. The released drug corresponded to 90% after 4 h and it was almost completely released (97%) after 7 h.

**Figure 9:**
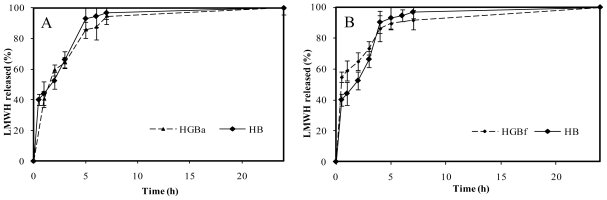
(A). Release profiles of low molecular weight heparin, LMWH, in PBS pH 7.4 from non crosslinked chitosan microspheres (HB) and with the highest genipin crosslinking condition, HGBa (25 mM genipin, 15 h, 50 ºC). (B). Release profiles of LMWH in PBS pH 7.4 from non crosslinked chitosan microspheres (HB) compared with the slightest crosslinking condition, HGBf (2.5 mM genipin, no thermal incubation).

Surprisingly, no significant differences between genipin crosslinked microspheres (HGB) and non crosslinked microspheres (HB) were registered in the case of high crosslinking degree. Figure 9A illustrates the HGBa (25 mM genipin, 15 h, 50 ºC) drug release profile as an example. As it can be seen, stronger crosslinking conditions were not associated with more control over LMWH release. This fact could be related to the polymerization processes between genipin monomers. As Mi and co-workers proposed [20], polymerized genipin chains could lead to wider nets in the microsphere matrix and, as a consequence, to less control in drug release.

On the other hand, LMWH release kinetics corresponding to slight crosslinking conditions did not lead to higher control in the delivery of the drug in comparison with non crosslinked microspheres, HB. An example is shown in Figure 9B, corresponding to HGBf microspheres (2.5 mM genipin, without thermal incubation). In this case, the absence of control over the drug release in comparison with the non crosslinked microspheres could be explained because of an insufficient degree of crosslinking.

**Figure 10:**
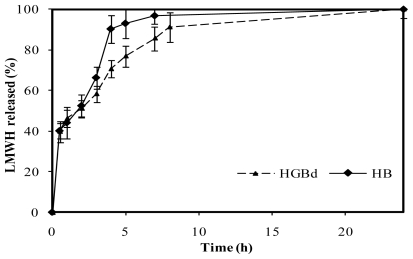
Release profiles of low molecular weight heparin, LMWH, in PBS pH 7.4 from non crosslinked chitosan microspheres and genipin crosslinked microspheres HGBd (0.5 mM genipin, 5 h, 50 ºC).

In contrast, medium crosslinking conditions, corresponding to 0.5 mM genipin incubated during 5 h at 50 ºC (named HGBd), led to a more controlled release compared with HB. These were the only crosslinking conditions that led to LMWH controlled release within those assayed in this study, summarized in Table 3. As shown in Figure 10, following a burst effect, LMWH release increased from 40% to 60% after 3 h in a similar way to that described for HB. Nevertheless, the drug release to the medium was slower throughout the period 4–8 hours. After 4 h, less than 70% of the LMWH had been released from medium crosslinked microspheres, which is significantly lower than 90% released from HB. Finally, 90% LMWH was released from HGBd after 8 hours, reaching 100% within the period 8–24 hours.

The release profile registered with this drug delivery model was comparable with those obtained by other authors with different LMWH delivery systems. Thus, our results showed higher control in the release than that obtained by some authors [21], but retained the drug to a less extent than others [22–24].

## 3. Experimental Section

Chitosan hydrochloride (HCS) was obtained from Novamatrix (Norway). The degree of deacetylation was 86% and the molecular weight (Mw) was 400 KDa. Clarithromycin and tramadol hydrochloride were provided by Vegal Farmacéutica S.L. (Spain), LMWH was supplied by Laboratorios ROVI S.A. (Madrid, Spain) and genipin was purchased from Challenge Bioproducts Co., LTD. All other reagents were commercially available and used without any modification.

### 3.1. Characterization of the crosslinking reaction

A 5 mg/mL chitosan solution was prepared by disolving it in deionized water. Genipin solutions of different concentrations (0–5 mM) were added and the resulting mixture was incubated for different time intervals (30 min–7 h) to allow the crosslinking to occur, at 50 ºC. The chitosan-genipin crosslinking reaction was characterized by UV/Vis (GBC UV/Vis 920, GBC Scientific Equipment Pty. Ltd., Dandenong, Australia). During the crosslinking reaction, spectra were obtained at certain time intervals in a wavelength range from 200 to 700 nm, at 2 nm resolution.

### 3.2. Preparation of drug loaded chitosan-genipin microspheres

The required amount of the model drug with respect to polymer weight (50% w/w of clarithromycin, 30% w/w of tramadol hydrochloride, 10% w/w of LMWH) was added to the chitosan solution (5 mg/mL in the case of clarithromycin and tramadol hydrochloride and 1mg/mL for LMWH) with constant stirring. Genipin solution was added to the chitosan solution in different concentrations (0–25 mM) and the resulting mixture was incubated at 50 ºC during different time periods (0 and 15 h). Drug/polymer concentration, reaction temperature, and genipin concentration ranges used in each case are based on previous investigations carried out in our laboratory (data not shown). Chitosan-genipin-drug solutions were spray-dried using a Büchi Mini Spray Dryer B-290 (Switzerland) with a standard 0.5 mm nozzle. Briefly, spray-drying conditions were inlet temperatures of 150 ºC for LMWH and 160 ºC for clarithromycin and tramadol hydrochloride, spray flow rate of 473 NL/h, aspirator air flow rate of 32 m^3^/h and sample flow rate of 2.5 mL/min.

### 3.3. Morphology

To determine shape and surface of the microspheres scanning electron microscopy (SEM) was used. The samples were sputter coated with Au/Pd using a vacuum evaporator (Balzers SDC 004 Sputter coater, Oerlikon Corporate Pfäffikon, Switzerland), and examined using a scanning electron microscope (JEOL JSM-6400 (JEOL, Tokyo, Japan) at 10 kV accelerating voltage.

### 3.4. Zeta potential

Microspheres were suspended in ethanol. A solution of 10^−3^ M KCl was prepared. 1 mL of microsphere suspension was diluted into 99 mL KCl solution. Zeta potential measurements were made by microelectrophoresis using a Malvern Zetasizer Nanoseries Nano ZS (Malvern Instruments, Herrenberg, Germany). Measurements were performed at an effective voltage of 150 V and 25 ºC. Electrophoretic mobility data were automatically converted into zeta potential using the Henry equation and the approximation of Helmholtz-Smoluchowski [25].

### 3.5. X-ray diffraction

X-ray diffraction patterns were obtained using an X-ray diffractometer (PHILIPS X’PERT SW) with a copper anode. The samples were scanned continuously from 0º to 50º (2θ) at 45 kV and 40 mA.

### 3.6. In vitro drug release

Microspheres inside a cellulose dialysis bag (dialysis tubing, Mw cut off 12,000 Da, Sigma Aldrich, Madrid, Spain) to avoid the loss of microspheres during the experiment, were suspended in HCl 1N pH 1.2 as simulated gastric fluid, SGF (clarithromycin and tramadol hydrochloride) or PBS pH 7.4 (LMWH) without enzyme. The dialysis bag was placed in a glass with 50 mL (clarithromycin), 200 mL (tramadol hydrochloride) or 100 mL (LMWH) of the release medium at 37 ºC with orbital agitation of 100 rpm (Rotabit horizontal shaker, Selecta, Barcelona, Spain). The volume of the release recipient was established on the basis of previous studies for each drug, so that the release medium would not be saturated. At predetermined interval times, 2 mL of sample were withdrawn. The withdrawn volume was replaced with fresh medium at the same temperature. The drug released was quantified by HPLC in the case of clarithromycin and UV/Vis in the case of tramadol hydrochloride and LMWH. All the experiments were carried out in triplicate.

## 4. Conclusions

The results of this study support the relevance of genipin as a valuable natural, non toxic, crosslinking agent for controlled drug release in drug delivery systems such as chitosan hydrochloride microspheres. Genipin-chitosan delivery systems have shown to be useful to control the release of such different drugs as clarithromycin, tramadol hydrochloride and low molecular weight heparin loaded in spray-dried microspheres. Nevertheless, for pharmaceutical applications, particular crosslinking conditions should be studied in each case depending on the nature of the drug, dose, release location in the human body, and pharmacokinetic factors, among others. Moreover, *in vivo* studies are needed to support the use of genipin for controlled drug release.

## Figures and Tables

**Table 1:** Zeta potential (mV) of chitosan hydrochloride (5 mg/mL) microspheres with 50% (w/w) clarithromycin without genipin (C1) and crosslinked with 0.5 mM genipin (C2) and 1 mM genipin (C3) at 50 ºC for 5 h.

Microspheres	Genipin mM	Crosslinking time (hours)	Zeta Potential mV
C1	0	0	49.3 ± 5.4
C2	0.5	5	38.6 ± 1.10
C3	1	5	32.9 ± 2.15

**Table 2:** Zeta potential (mV) of chitosan hydrochloride (5 mg/mL) microspheres with 30% (w/w) tramadol hydrochloride without genipin (T1) and crosslinked with 2 mM genipin (T2) and 5 mM genipin (T3) at 50 ºC for 5 h.

Microspheres	Genipin mM	Crosslinking time (hours)	Zeta Potential mV
T1	0	0	24.6 ± 3.15
T2	2	5	14.84 ± 1.06
T3	5	5	14.50 ± 0.46

**Table 3:** Zeta potential (mV) of chitosan hydrochloride (1 mg/mL) microspheres (HCS), chitosan hydrochloride-LMWH microspheres (HB) and chitosan hydrochloride-LMWH microspheres, crosslinked with different genipin concentration (HGBa-HGBf) at 50 ºC for 0, 5 or 15 h.

Microspheres	Genipin mM	Crosslinking time (hours)	Zeta Potential mV
HCS	0	0	25.51 ± 0.69
HB	0	0	23.04 ± 0.96
HGBf	2.5	0	19.95 ± 0.99
HGBd	0.5	5	14.68 ± 2.52
HGBc	0.5	15	12.21 ± 0.76
HGBb	12.5	15	10.36 ± 0.84
HGBa	25	15	7.42 ± 0.23
